# Effective Degradation of Aqueous Tetracycline Using a Nano-TiO_2_/Carbon Electrocatalytic Membrane

**DOI:** 10.3390/ma9050364

**Published:** 2016-05-12

**Authors:** Zhimeng Liu, Mengfu Zhu, Zheng Wang, Hong Wang, Cheng Deng, Kui Li

**Affiliations:** 1 Institute of Medical Equipment, Academy of Military Medical Sciences, Tianjin 300161, China; liuzhimeng89@126.com (Z.L.); 18801024666@163.com (Z.W.); likui2013@126.com (K.L.); 2 State Key Laboratory of Hollow Fiber Membrane Materials and Processes, School of Materials Science and Engineering, Tianjin Polytechnic University, Tianjin 300387, China; waho7808@163.com

**Keywords:** electrocatalytic membrane, nano-TiO_2_, electrocatalytic oxidation, membrane separation, tetracycline

## Abstract

In this work, an electrocatalytic membrane was prepared to degrade aqueous tetracycline (TC) using a carbon membrane coated with nano-TiO_2_ via a sol-gel process. SEM, XRD, EDS, and XPS were used to characterize the composition and structure of the electrocatalytic membrane. The effect of operating conditions on the removal rate of tetracycline was investigated systematically. The results show that the chemical oxygen demand (COD) removal rate increased with increasing residence time while it decreased with increasing the initial concentration of tetracycline. Moreover, pH had little effect on the removal of tetracycline, and the electrocatalytic membrane could effectively remove tetracycline with initial concentration of 50 mg·L^−1^ (pH, 3.8–9.6). The 100% tetracycline and 87.8% COD removal rate could be achieved under the following operating conditions: tetracycline concentration of 50 mg·L^−1^, current density of 1 mA·cm^−2^, temperature of 25 °C, and residence time of 4.4 min. This study provides a new and feasible method for removing antibiotics in water with the synergistic effect of electrocatalytic oxidation and membrane separation. It is evident that there will be a broad market for the application of electrocatalytic membrane in the field of antibiotic wastewater treatment.

## 1. Introduction

In recent years, the emergence of antibiotics in the environment has received increasing attention [1,2]. A large number of antibiotic residues are detected in the aquatic ecosystem due to the extensive production and use of antibiotics [3], and some reports even pointed out that the antibiotic residues were also detected in tap water. The presence of antibiotics in water not only affects the water quality, but also causes potential adverse effects on humans and ecological systems [4]. Although the concentration of antibiotics is very low in the environment (ng·L^−1^ or μg·L^−1^), the antibiotics are hard to be degraded by microorganism due to the complex structure and the antibacterial nature [5]. Hence, antibiotics are gradually enriched in the environment resulting in antibiotic pollution. When long-term exposure to an antibiotic-polluted environment, human health would be affected even at very low concentrations [6]. Among all of the antibiotics, tetracycline (TC) is wildly used in human and veterinary medicine [7]. TC has been detected in sewage water, surface water, groundwater, drinking water, and sludge due to its ineffective removal by conventional water treatment processes [8,9,10].

At present, the conventional methods to treat antibiotic wastewater are physical methods (coagulant sedimentation, adsorption, and membrane separation) [11], biological methods (activated sludge, biological contact oxidation, anaerobic sludge bed) [12], advanced oxidation processes (AOPs) (ozone oxidation, fenton oxidation, photacatalytic oxidation, electrocatalytic oxidation) [13,14,15], and some combination methods [16,17,18]. However, the traditional physical and biological methods cannot remove TC effectively. Membrane separation technology (mainly Nanofiltration (NF) and Reverse Osmosis (RO)) could remove TC from water [19], while membrane separation technology is based on the physical screening, which would inevitably result in membrane fouling and reduce the membrane flux. Although antibiotics could be removed by physical adsorption processes such as activated carbon [20], they were just transferred to another medium, which required further disposal. Owing to the biological toxicity of TC, inhibiting the microbial activity, the biological treatment methods cannot decompose TC effectively either.

The traditional electrochemical treatment processes are not suitable for aqueous TC treatment due to the low efficiency and high energy consumption. Electrocatalytic oxidation is a novel AOPs method utilizing the catalyst to enhance the electrochemical reaction. The organic compounds are degraded by the hydroxyl radical (OH) and other active radicals generated by electrocatalysis, and no additional chemicals are needed [21]. During the whole electrocatalytic oxidation process, no oxidation byproducts are generated. Therefore, the electrocatalytic oxidation of recalcitrant organic pollutants such as antibiotics have attracted increasing interest in recent years. Zhang *et al.* designed the TC degradation experiment by anode oxidation with a Ti/RuO_2_-IrO_2_ electrode and investigated the operating parameters such as electrical current density, initial pH, and antibiotic initial concentration on the TC oxidation effect [22]. Further, Nihal Oturan systematically investigated the effect of different cathode materials (carbon-felt and stainless steel) and anode materials (Ti/RuO_2_-IrO_2_, Pt, and BDD) on the direct/indirect electro-oxidation of TC [23].

However, compared with the membrane separation process, these usual electrocatalytic processes are operated intermittently, which limit the increase of total water flow and hinder the extensive application of electrocatalytic oxidation technology in water treatment field. Li *et al.* introduced electrocatalytic oxidation into the membrane separation process and designed an electrocatalytic membrane reactor (ECMR) with a self-cleaning function and continuous operation for wastewater treatment [24]. In their work, a tubular conductive membrane with nano-TiO_2_ loading as the anode and a stainless steel mesh as a cathode constituted an ECMR. Once the membrane anode is electrified, excitation of TiO_2_ creates electron-hole pairs. The obtained electrons and holes react with the H_2_O and O_2_ to generate reactive intermediates such as ·OH, ·O_2_-, ·HO_2_, and H_2_O_2_. These reactive intermediates can indirectly decompose the organic pollutants located on the surface or in the pores of the membrane into CO_2_ and H_2_O, or other biodegradable products. Therefore, both the membrane separation and electrocatalytic oxidation function can be achieved through the electrocatalytic membrane technology. Subsequently, Yang used ECMR to treat 200 mg·L^−1^ oily wastewater. The results showed that the chemical oxygen demand (COD) removal rates were up to 87.4 and 100 with the liquid hourly space velocity of 7.2 h^−1^ and 21.6 h^−1^, respectively [25]. Wang and co-workers found the phenol removal rate and complete mineralization fraction were 99.96% and 72.4%, respectively, when treated by ECMR under the conditions of 10.0 mM phenolic wastewater, pH of 6, current density of 0.3 mA·cm^−2^, and residence time of 5.2 min [26]. Liu reported that using carbon nanotubes (CNT) as the anode, an electrochemical filter was fabricated, which could treat aqueous tetracycline effectively. The tetracycline oxidative flux was 0.025 ± 0.001 mol·h^−1^m^−2^ at an initial tetracycline concentration of 0.2 mM, total cell potential of 2.5 V, and flow rate of 1.5 mL·min^−1^ [27]. All of these demonstrate that the electrocatalytic membrane is a highly efficient water treatment method.

The purpose of this work was to develop a novel process to treat aqueous TC by an electrocatalytic membrane. The electrocatalytic membrane was fabricated by a carbon membrane coated with nano-TiO_2_ via a sol-gel process. With the synergistic effect of electrocatalytic oxidation and membrane separation, the nano-TiO_2_/carbon composite membrane could remove aqueous TC efficiently. COD and TC concentration were employed as the detection index to illustrate the efficiency of TC degradation. The operating parameters, such as current density, temperature, residence time, initial TC concentration, and pH for the influence of TC degradation efficiency were analyzed systematically.

## 2. Results and Discussion

### 2.1. Composition and Structure of Nano-TiO_2_/Carbon Membrane

The detailed specifications of the nano-TiO_2_/carbon membrane including thickness, area, average pore size, porosity, and electrical resistivity are presented in Table 1.

**Figure 1:**
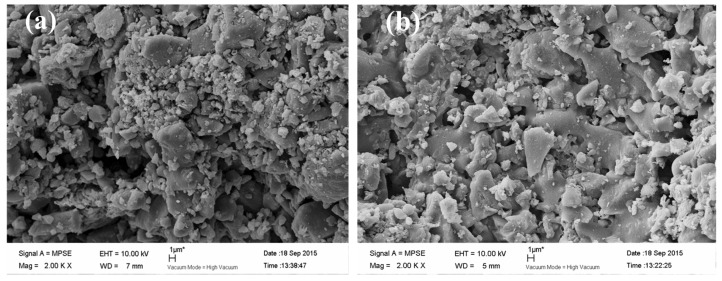
SEM images of the original carbon membrane (**a**) and nano-TiO_2_/carbon membrane (**b**).

To observe the morphological structure of original carbon membrane and nano-TiO_2_/carbon membrane, SEM analysis was performed (Figure 1). As shown in Figure 1a, the original carbon membrane surface is extremely rough and possesses a channel structure of irregular shape, which is conducive to improve the specific surface area and benefit the adsorption of organic molecules. It is generally known that the larger specific surface, the better efficiency of the catalytic reaction. A large amount of carbon particles heaped on the membrane surface which will be favorable for loading TiO_2_ and getting a larger effective catalytic area, further increasing the catalytic efficiency of the electrocatalytic membrane. According to Figure 1b, the nano-TiO_2_/carbon membrane maintained its original rich porous structure, which contributes to its membrane separation function. However, no TiO_2_ particles are observed obviously on the membrane surface by SEM analysis.

To further confirm the existence of TiO_2_ on the prepared composite ceramic membrane, EDS analysis was conducted. The results are shown in Table 2. For the original carbon membrane, it is evident that there was no titanium on the original carbon membrane, while for the nano-TiO_2_/carbon membrane, it was found that the content of titanium on the surface and cross-section was 12.45 and 5.24 wt %, respectively. This shows that the dip-coating method is good for the sol getting into the internal microporous structure of the membrane. The content of titanium on cross-section was less than that on the surface, which illustrates that the membrane surface has a higher catalytic efficiency and pollutants are more likely to be degraded on the membrane surface.

**Figure 2:**
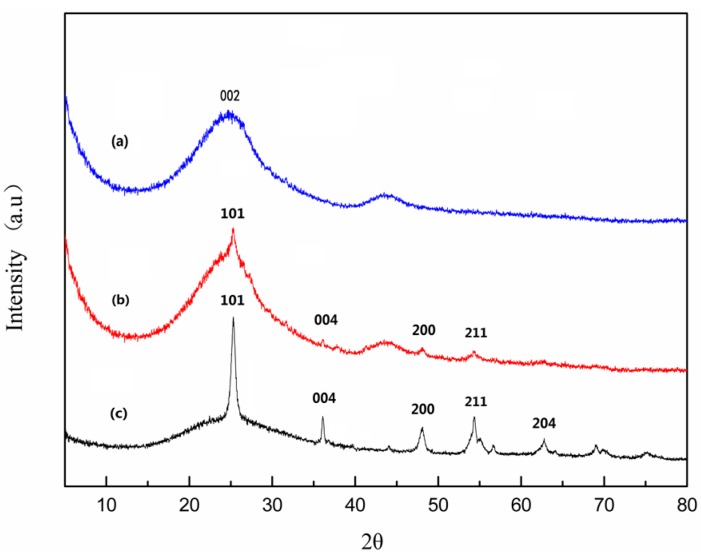
XRD patterns of the original carbon membrane (**a**); nano-TiO_2_/carbon membrane (**b**); and synthesized TiO_2_ (**c**).

The chemical composition of the membrane surface changed after being coated with nano-TiO_2_. To study the chemical structure of membrane surface, XRD (Figure 2) analysis were performed. As shown from XRD analysis of the original carbon membrane (Figure 2a), there has a broad weak (002) peak which is attributed to the graphitization structure generated after the process of calcining. It can also be seen from the spectrum of synthesized TiO_2_ (Figure 2c) that the diffraction peaks at 25.37°, 37.87°, 48.11°, 55.10°, and 62.73° belong to cubic TiO_2_ (101), (004), (200), (204), and (211) crystal planes, respectively, which reveals the crystal phase is in the anatase phase. In Figure 2b, the diffraction peaks from the nano-TiO_2_/carbon membrane is relatively consistent with that from the anatase TiO_2_ (Figure 2c). Owing to the low content of Ti element in the membrane, the characteristic diffraction peaks of TiO_2_ are weak, in good agreement with the EDS analysis above. Thus, the XRD result indicates that the nano-TiO_2_/carbon membrane was prepared successfully via nano-TiO_2_ surface modification.

**Figure 3:**
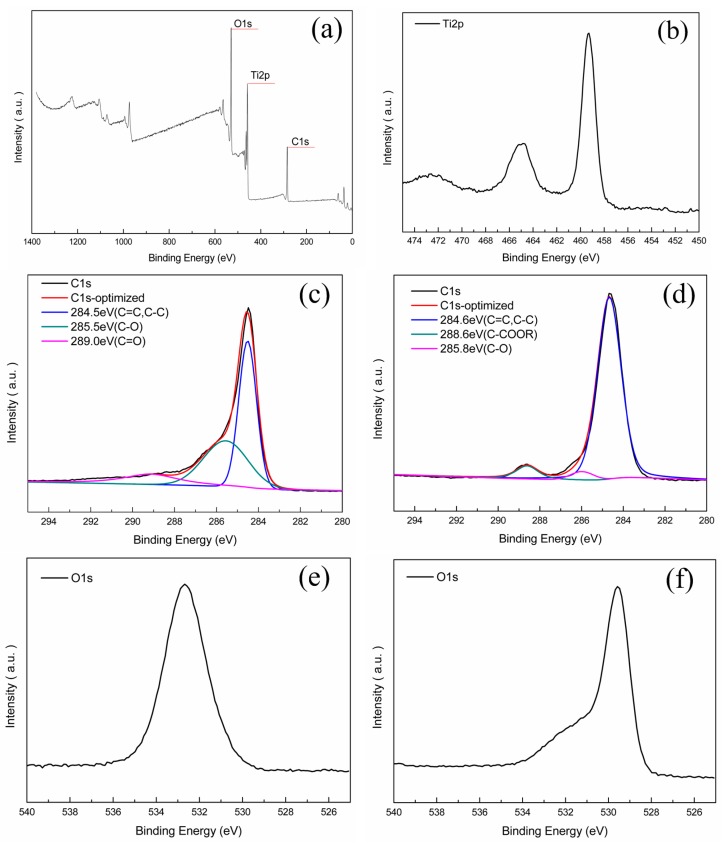
XPS spectra of the samples: (**a**) survey spectra of the nano-TiO_2_/carbon membrane; (**b**) Ti 2*p* region of the nano-TiO_2_/carbon membrane; (**c**) C 1*s* region of the original carbon membrane; (**d**) C 1*s* region of the nano-TiO_2_/carbon membrane; (**e**) O 1*s* region of the original carbon membrane; and (**f**) O 1*s* region of the nano-TiO_2_/carbon membrane.

XPS provided convincing evidences of nano-TiO_2_ coating on carbon membrane and gave the composition information of the original and nano-TiO_2_/carbon membrane (Figure 3). The survey spectrum (Figure 3a) shows clear constituents of element C, O, and Ti. Figure 3b displays that the Ti 2*p* peaks of the nano-TiO_2_/carbon membrane were at 454.9 and 465.1 eV, which are attributed to the Ti–O bond [28]. To be particular, though, the high-resolution C 1*s* spectra (Figure 3c,d) show a similar distribution of signals, the intensity of each signal differs observably. The strongest signal of the original carbon membrane emerged at 285.5 eV attributing to C–O bond (Figure 3c) and that of the nano-TiO_2_/carbon membrane was found at 285.7 eV (Figure 3d). However, the C–O intensity of the nano-TiO_2_/carbon membrane was weaker than that of the original carbon membrane. This is likely due to the TiO_2_ coating on the carbon membrane causing most of the oxygen element forming Ti–O bonds. Hence, the C–O intensity of the nano-TiO_2_/carbon membrane decreased compared with that of original carbon membrane. From the O 1*s* spectrum of the original carbon membrane (Figure 3e) and the nano-TiO_2_/carbon membrane (Figure 3f), an obvious shift from the original carbon membrane at 532.67 eV to the nano-TiO_2_/carbon membrane at 529.58 eV could be observed. The emission peak at 529.58 eV was in accord with that of pure TiO_2_ at 529.6 eV [29], illustrating that the majority of oxygen formed a Ti–O bond. The result also explains why the intensity of the C–O bond of the nano-TiO_2_/carbon membrane decreased.

### 2.2. Performance of Nano-TiO_2_/Carbon Membrane in Degradation of TC Solution

#### 2.2.1. Effect of Current Density

**Figure 4:**
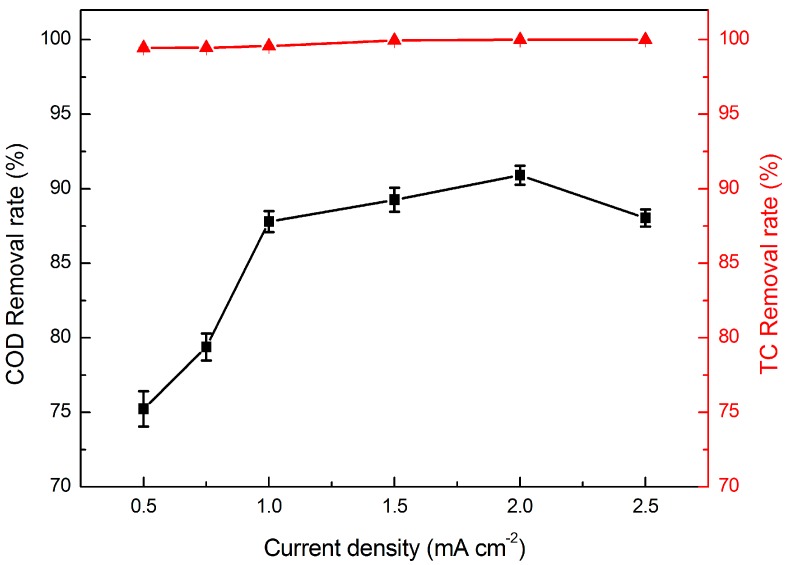
Effect of current density on the removal rate of TC (▲) and COD (■). (*TC* = 50 mg·L^−1^, *RT* = 4.4 min, and *T* = 25 °C).

The effect of current density on the degradation of TC was investigated at different current densities when the TC concentration was 50 mg·L^−1^, the residence time was 4.4 min, and the temperature was 25 °C. As shown in Figure 4, the TC removal rate was always greater than 99%, which explicates that the molecular structure of TC was destroyed by electrocatalytic membrane oxidation decomposition. Thus, TC was effectively removed in permeate liquid. Observing the COD removal rate in Figure 4, it is obvious that the COD removal rate was less than that of the TC removal rate. This is mainly because that the intermediate products of the TC molecules are not completely decomposed or not completely mineralized. The COD removal rate increased with the increase of current density. The COD removal rate was 75.23% and 90.91% when the current density of 0.5 and 2 mA·cm^−2^, respectively. This is ascribed to the fact that, at high current density, the speed of the electron transfer increased, resulting in a greater amount of reactive oxidizing groups, such as ·OH, in unit time. Therefore, it will be advantageous to the degradation of organic molecules. Instead, the COD removal rate declined when the current density increased to 2.5 mA·cm^−2^; this is because the high current density will promote the secondary reaction, such as the oxygen evolution reaction, and make the electrode surface hydrolyse to produce oxygen which hinders the organic matter in contact with the electrode surface. Thus, the COD removal rate will decrease if the current density is too high. On the other hand, the energy consumption will increase with the increase of current density. Therefore, the best current density of electrocatalytic membrane equipment was selected as 1 mA·cm^−2^ in order to reduce energy consumption and obtain high removal efficiency.

#### 2.2.2. Effect of Temperature

**Figure 5:**
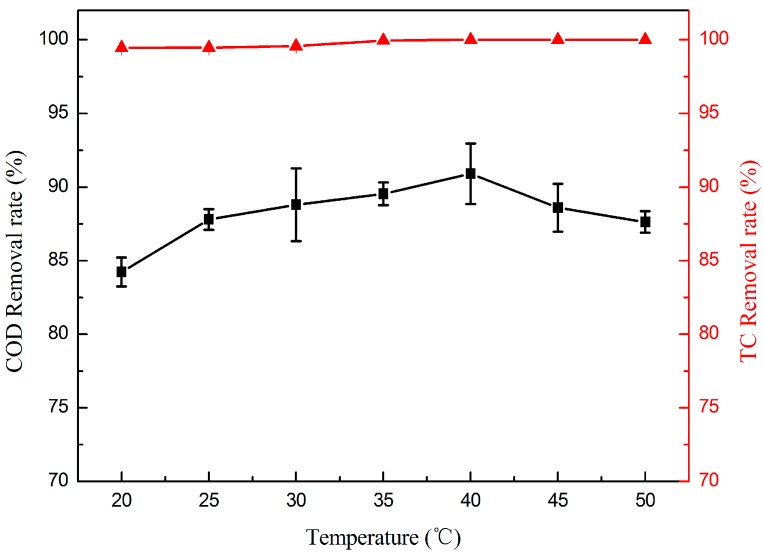
Effect of temperature on the removal rate of TC (▲) and COD (■). (*TC* = 50 mg·L^−1^, *RT* = 4.4 min and *j* = 1 mA·cm^−2^).

The oxidation efficiency of the electrode could be influenced greatly by the reaction temperature. The effect of temperature on TC degradation was studied from 20 to 50 °C. In Figure 5, it is clearly observed that the COD removal rate increased in the first stage, and then decreased with the increasing temperature, while the TC removal rate of nearly 100% could be achieved at all of the tested temperatures. Raising the temperature can decrease the solution viscosity and increase the mass transport rate from solution to the membrane surface, which will promote TiO_2_ excitation to generate more electrons and holes; thus, much more ·OH will be produced to degrade the TC as well as its intermediates. However, the ability of TiO_2_ to generate electrons and holes was close to maximum when the temperature rose to a certain degree. The COD removal rate decreased with increasing temperature, which could be attributed to the oxygen evolution reaction. The oxygen evolution reaction intensified at higher temperature, which induced the electrode surface to be covered by a large number of bubbles, furthermore decreasing the electrode effective area and mass transport efficiency.

#### 2.2.3. Effect of Residence Time

**Figure 6:**
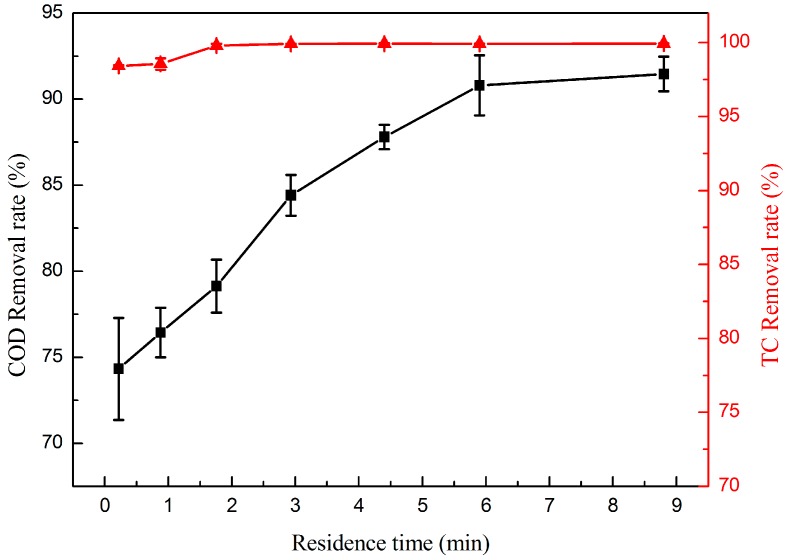
Effect of residence time on the removal rate of TC (▲) and COD (■). (*TC* = 50 mg·L^−1^, *T* = 25 °C, and *j* = 1 mA·cm^−2^).

In order to investigate the efficiency of the electrocatalytic membrane for TC and COD removal, the filtration performance test was carried out under the residence time from 0.22 to 8.8 min during the TC solution of 50 mg·L^−1^. The effect of residence time on the removal of TC and COD is shown in Figure 6.

The TC removal rates were more than 98% when the residence time ranged from 0.22 to 8.8 min, which indicates the electrocatalytic membrane can efficiently destroy the molecular structure of TC in a short time. Moreover, TC removal rates could be close to 100% when the residence time increased to 1.8 min. The COD removal rate increased along with the extension of residence time and it could be up to 91.46% when the residence time increased to 8.8 min. All of these results illustrates that the longer the residence time, the more complete the degradation of the TC. This is because the longer contact time of contaminants and the electrocatalytic membrane means increasing the electrocatalytic reaction time and, consequently, the degradation reaction will be more thorough. However, if the residence time was too long, the declined mass transport efficiency of the solution will lead to the decreasing TC degradation in unit time and high energy consumption. The electrocatalytic membrane could achieve the removal efficiencies of nearly 100% TC and 87.8% COD for residence time of 4.4 min, respectively. So, considering the energy consumption and efficiency of the removal rate of TC, the residence time was selected as 4.4 min.

#### 2.2.4. Effect of TC Initial Concentration

**Figure 7:**
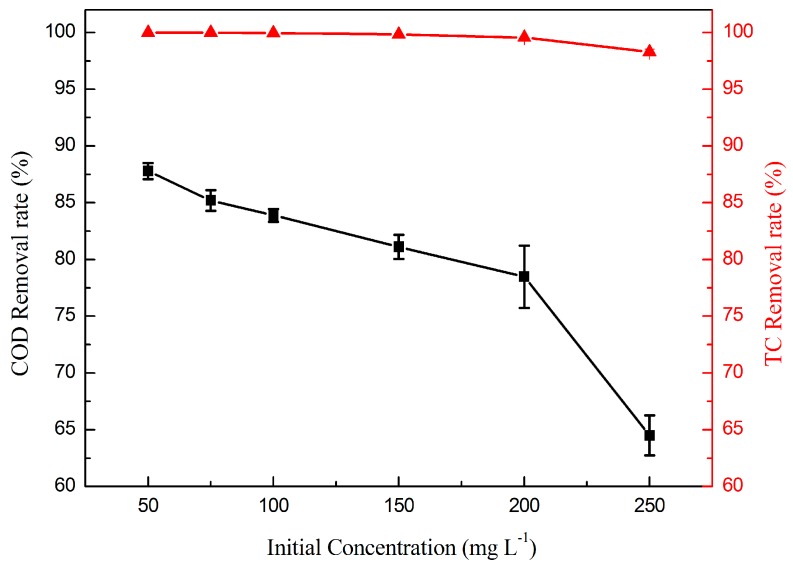
Effect of initial concentration on the removal rate of TC (▲) and COD (■). (*RT* = 4.4 min, *T* = 25 °C, and *j* = 1 mA·cm^−2^).

The initial concentration of the reactants often affects mass transport resistances and reaction efficiency. Hence, six different TC initial concentrations (50, 75, 100, 150, 200, and 250 mg·L^−1^) were tested to investigate the degradation performance. Figure 7 illustrates the removal efficiency of TC at different initial concentrations. As can be seen, the removal efficiency of COD decreased with increasing TC initial concentration. The highest COD removal rate was 87.8%, obtained at 50 mg·L^−1^, while the lowest COD removal rate was 64.5%, obtained at 250 mg·L^−1^. The TC removal rate was more than 99% when the concentration was below 200 mg·L^−1^, and the TC removal rate decreased slightly once the concentration exceeded 200 mg·L^−1^. Due to the same membrane volume and the same operating conditions of the electrocatalytic membrane, the amounts of generated ·OH should be fixed. Thus, the limited removal capability cannot break the molecular structure of TC and its intermediates at the higher initial concentration. Consequently, the COD removal rate decreased with the increasing TC concentration.

#### 2.2.5. Effect of pH

**Figure 8:**
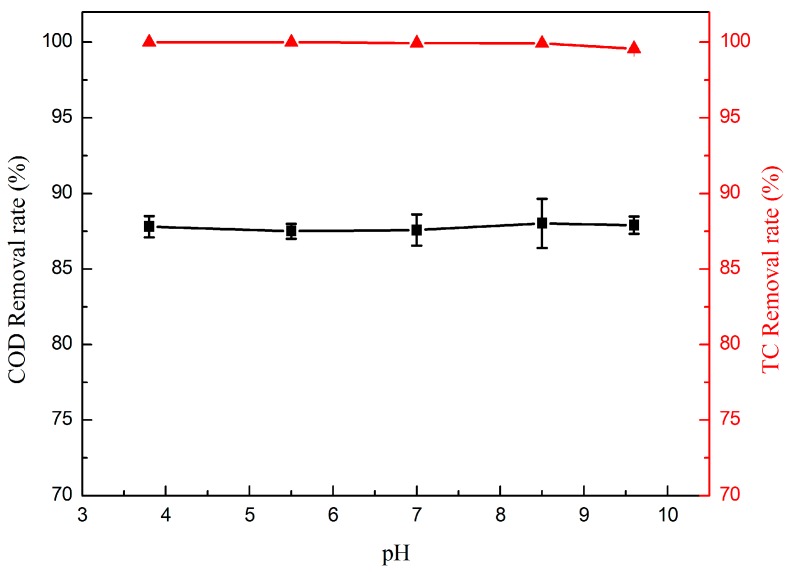
Effect of pH on the removal rate of TC (▲) and COD (▲). (*TC* = 50 mg·L^−1^, *RT* = 4.4 min, *T* = 25 °C, and *j* = 1 mA·cm^−2^).

The pH of the solution is the factor affecting the performance of the electrocatalytic oxidation process. In order to elucidate if TC could be degraded effectively in a wide pH range, a series of filtration experiments were performed, using 50 mg·L^−1^ TC for initial pH of 3.8, 5.6, 7, 8.5 and 9.6, respectively. As shown in Figure 8, there is no significant difference for the COD and TC removal rate at different pH values, and the COD and TC removal rates are almost constant with a high efficiency no matter what the pH is. This result indicates that the electrocatalytic membrane could perform efficiently at any pH value between 3.8 and 9.6. The nano-TiO_2_/carbon electrocatalytic membrane has good stability in a wide pH range and will certainly have a broad application range and application prospects.

#### 2.2.6. Effect of Operation Time

**Figure 9:**
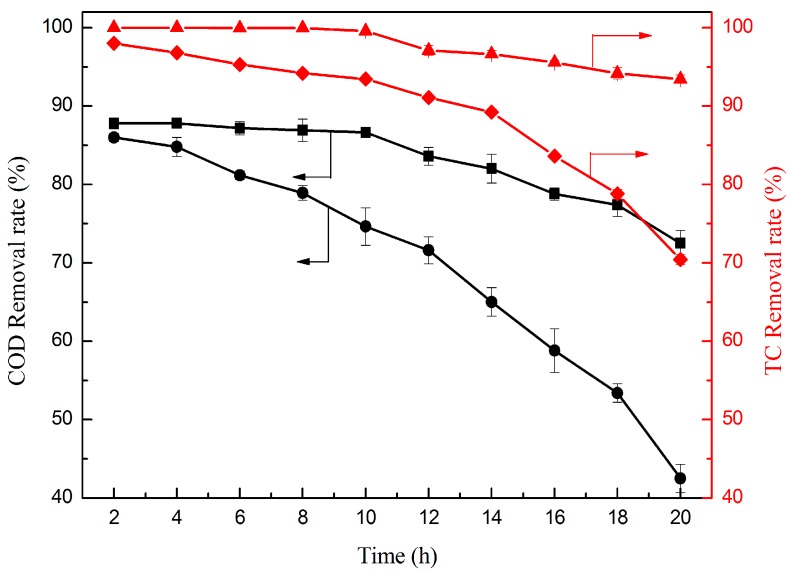
TC removal rate by the TiO_2_/carbon membrane (▲) and the original carbon membrane (◆); and COD removal rate by the TiO_2_/carbon membrane (■) and the original carbon membrane (●) *versus* operation time. (*RT* = 4.4 min, *T* = 25 °C, and *j* = 1 mA·cm^−2^).

To further evaluate the stability of the electrocatalytic membrane and the contribution of TiO_2_, the long-time operation and the control experiments of the original carbon membrane for treating TC *versus* operation time was conducted (Figure 9). As the result shows, the TC removal rate of the TiO_2_/carbon membrane could always maintain a high removal rate. Nearly 100% TC removal rate could be achieved in the first 10 h and decrease slowly when the operation time exceeded 10 h. By contrast, the TC removal rate of the original carbon could also maintain a high removal rate during the initial period, while much lower than that of the TiO_2_/carbon membrane with the increase of the operation time. The COD removal rate was obviously different by means of the original carbon membrane and the TiO_2_/carbon membrane, namely, the COD removal rate of the original carbon membrane decreased much faster over the operation time than that of the TiO_2_/carbon membrane. The reason is that an increasing number of TC molecules accumulated on the membrane with the flow, however, the oxidative degradation ability of the TiO_2_/carbon membrane is not capable enough to degrade TC molecules completely, resulting in some molecules passing through the membrane. Hence, the TC and COD removal rate decreased with increasing operation time. The original carbon membrane is a good conductor, which could be used as an electrochemical electrode. The TC molecules on the membrane could be decomposed into micromolecular intermediates, or even CO_2_ and H_2_O by anodic oxidation. As a result, the original carbon membrane has a certain removal rate of TC as well. However, the TiO_2_/carbon membrane has the larger specific surface area due to the nano-TiO_2_ coating on the membrane, which increases the reaction area. Moreover, the TiO_2_ could be excited to generate reactive intermediates such as ·OH, O_2_^−^, and HO_2_, which can decompose the organics located on the membrane more completely [26]. Therefore, the TiO_2_/carbon membrane is of high removal efficiency and high stability for TC degradation and has the better effect on both TC and COD removal rate than the original carbon membrane.

## 3. Experimental Section

### 3.1. Materials and Reagents

The original carbon membrane was obtained from Dalian University of Technology, which was prepared through a sintering process. First, a certain amount of adhesive was mixed into the coal powder and phenolic resin powder, then was pressed and molded into a uniform flat mold. After drying, the flat mold was further placed in the high-temperature carbonization furnace, and then the flat carbon membrane was obtained [30]. TC hydrochloride (C_22_H_24_N_2_O_8_·HCl) (96% purity) was purchased from Macklin (Macklin Biochemical Co., Ltd., Shanghai, China). All the other reagents from Kermel (Kermel Chemical Reagent Co., Ltd., Tianjin, China) were of analytically pure grade (AR) and used without further purification.

### 3.2. Preparation and Characterization of the Nano-TiO_2_/Carbon Membrane

The square carbon membrane was first pretreated in 65 wt % HNO_3_ solution for 30 min, cleaned with deionized water, and dried for 100 min at 80 °C. Tetrabutyltitanate, anhydrous ethanol, deionized water, glacial acetic acid, and diethanolamine were added to the flask at certain proportions sequentially. After strong stirring for 120 min and aging 24 h at room temperature, the tetrabutoxide sol could be achieved. The activated carbon membrane was dipped into the tetrabutoxide sol for 30 min, then removed from the solution and dried at room temperature. Finally, the treated membrane was placed in a muffle furnace to sinter at 430 °C for 120 min to prepare the nano-TiO_2_/carbon membrane [24].

Powder X-ray diffraction (XRD; MiniFlex600, Rigaku, Japan) was adopted to analyze the composition and crystal structure of the membrane [31]. Chemical bond analyses were obtained using X-ray photoelectron spectroscopy (XPS; VG ESCALAB MK II Instrument, East Grinstead, Sussex, UK) [32,33]. Samples were crushed to powders with agate mortar and pestle. Moreover, the microstructural analyses of the nano-TiO_2_/carbon membrane surface were characterized using scanning electron microscopy (SEM; LEO 1530VP, Oberkochen, Germany) in combination with energy dispersive spectroscopy (EDS; Trident, EDAX, Mahwah, NJ, USA), which enabled elemental analyses of microstructures.

### 3.3. Evaluation of TC Removal Performance

#### 3.3.1. Electrocatalytic Membrane Assembly Filter

**Figure 10:**
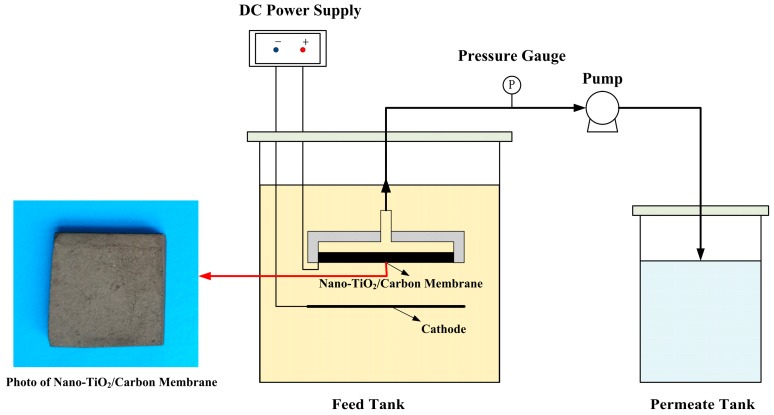
Schematic of the electrocatalytic membrane assembly filter.

A nano-TiO_2_ loading microporous carbon membrane as an anode, and a stainless steel mesh below the membrane, as a cathode were connected by a DC regulated power supply to constitute the electrocatalytic membrane filter equipment for the degradation of aqueous TC (Figure 10). The as-prepared nano-TiO_2_/carbon membrane was fixed and sealed in an organic glass module with a through-hole on the other side of the module. The hole on the module was connected to the peristaltic pump through the pipe. During the treatment process, the solution permeated through the membrane from the outside in a dead-end manner and the treated water was obtained from the inside. The degraded “clean water” can be obtained from the outlet of the peristaltic pump.

#### 3.3.2. TC Removal Performance

The experimental device of electrocatalytic membrane filter for TC treatment was designed as in Figure 10. The tetracycline aqueous solution was prepared by mixing TC hydrochloride and deionized water with different ratios, and sodium sulfate with the concentration of 15 g·L^−1^ was added as an electrolyte. The TC and COD removal rates were chosen as two key parameters to evaluate the degradation efficiency and were measured after 2 h operation. Current density, temperature, residence time, TC concentration, and pH were selected as the operating parameters in order to investigate the influence on the degradation of TC. Furthermore, long-running was implemented to evaluate the stability performance of the electrocatalytic membrane and the contribution of TiO_2_ coated on the carbon membrane.

The TC concentration was detected by using high-performance liquid chromatography (HPLC) (Waters 2695). The column used was an Agilent Extend-C18 and the mobile phase was 30% acetonitrile and 69% oxalic acid solution of 0.1 mol·L^−1^ with a flow rate of 1.0 mL·min^−1^. The TC removal efficiency was monitored by calculating removal rate *R_TC_* (1):

(1) $$R_{TC}=\left(1-\frac{TC}{TC_{0}}\right)\times 100\%$$

where *TC*_0_ and *TC* are the tetracycline concentration of the feed and permeate solutions (mg·L^−1^), respectively.

The chemical oxygen demand (COD) was measured with an ultraviolet and visible spectrophotometer (DR5000, Hach). The method involved a 2 mL simple digestion in the COD reactor for 120 min at 150 °C before examining the absorbance equivalent concentration. The COD removal rate (*R_COD_*) could be calculated using the following Equation (2):

(2) $$R_{COD}=\left(1-\frac{\mathrm{COD}}{{\mathrm{COD}}_{0}}\right)\times 100\%$$

where COD_0_ and COD are the COD of the feed and permeate solutions (mg·L^−1^), respectively.

In all electrocatalysis processes, the current density is the most important parameter for controlling the reaction rate for the oxidation of organic compounds, and the current density is defined as Equation (3):

(3) $$j=\frac{I}{A}$$

where *j* represents the current density (mA·cm^−2^), I represents the current (mA), and *A* represents the area of the membrane (cm^2^).

The residence time (*RT*) was the key parameter of the electrocatalytic membrane and affects the electrocatalytic membrane degradation efficiency and TC removal. Residence time associated with flow rate of TC solution can be calculated by Equation (4) [34]:

(4) $$RT=\frac{V_{0}\varphi }{v}\times 100\%$$

where *V*_0_ is the volume of electrode (cm^3^), φ is the porosity of membrane, and *v* is the volumetric flow rate of TC solution (mL·min^−1^).

## 4. Conclusions

The nano-TiO_2_/carbon electrocatalytic membrane system was designed to treat aqueous TC. With the synergistic effect of electrocatalytic oxidation and membrane separation, the nano-TiO_2_/carbon composite membrane could remove aqueous TC efficiently. The efficiency of TC degradation has been found to be dependent on the operating parameters such as current density, temperature, residence time, and initial concentration. The 100% TC and 87.8% COD removal rate could be achieved under following conditions: 50 mg·L^−1^ TC, current density of 1 mA·cm^−2^, temperature of 25 °C and residence time of 4.4 min. In addition, the electrocatalytic membrane system could continuously operate with high removal efficiency and high stability for TC degradation. In summary, this study provides a novel, reliable and feasible method for removing aqueous antibiotics, and there will be broad application prospects of electrocatalytic membrane in the field of antibiotics wastewater treatment.

## Figures and Tables

**Table 1:** Characteristics of nano-TiO_2_/carbon membrane.

Parameters	Value
Thickness, mm	5
Area, cm^2^	9
Average pore size, μm	0.41
Porosity, %	41.17
Electrical resistivity, mΩ·m	4.5

**Table 2:** EDS results of the original carbon membrane and nano-TiO_2_/carbon membrane.

Membrane	Element/wt %		
	C	O	Ti
Original carbon membrane	88.64	11.36	0
Nano-TiO_2_/carbon membrane surface	72.64	14.91	12.45
Nano-TiO_2_/carbon membrane cross-section	82.44	12.32	5.24

## Abbreviations

The following abbreviations are used in this manuscript:

AOPs	Advanced Oxidation Processes
ECMR	Electrocatalytic Membrane Reactor
COD	Chemical Oxygen Demand
SEM	Electron Microscopy
EDS	Energy Dispersive Spectroscopy
XRD	X-ray Diffraction
XPS	X-ray Photoelectron Spectroscopy
HPLC	High Performance Liquid Chromatography
RT	Residence Time
TC	Tetracycline
NF	Nanofiltration
RO	Reverse Osmosis
